# Evaluation of raw milk samples and vendor‐derived *Staphylococcus aureus* and *Coxiella burnetii* prevalence in dairy delicatessens in eastern Turkey

**DOI:** 10.1002/fsn3.4236

**Published:** 2024-06-05

**Authors:** Muhammed Furkan Kaplan, Ece Kaplan, Ali Raza, Mehtap Demirler, Alper Baran, Seyda Cengiz, Mehmet Cemal Adiguzel

**Affiliations:** ^1^ Department of Microbiology, Faculty of Veterinary Medicine Ataturk University Erzurum Turkey; ^2^ Veterinary Control Institute Republic of Türkiye Ministry of Agriculture and Forestry Erzurum Turkey; ^3^ Department of Veterinary Public Health, Faculty of Veterinary Medicine Ataturk University Erzurum Turkey; ^4^ Department of Microbiology, Milas Faculty of Veterinary Medicine Mugla Sitki Kocman University Mugla Turkey

**Keywords:** antimicrobial resistance, *Coxiella burnetii*, dairy delicatessen, milk, *Staphylococcus aureus*

## Abstract

This study investigated the prevalence, antimicrobial resistance, and genetic diversity of *Staphylococcus aureus*, as well as the detection and genetic relationship of *Coxiella burnetii* in retail milk samples and plastic bags from 25 local dairy delicatessens. Bacteriological culture, PCR, and Sanger sequencing were performed. Eleven percent of the samples were positive for *S. aureus*, none of which were methicillin‐resistant or enterotoxigenic. The rep‐PCR analysis revealed a high similarity between strains isolated from milk and bag samples from the same source. Moreover, 14% of the milk samples were positive for *C. burnetii*, which matched 100% of the reference strains in the GenBank.

## Graphical abstract

*Staphylococcus aureus* and *Coxiella burnetii* were detected in retail milk and plastic bags sold at local dairy delicatessens in Eastern Turkey. The employees in the delicatessens should be aware of the risk to public health. There is a pressing need for more effective implementation of national and international guidelines to decrease the contamination of raw milk and vendor origins.

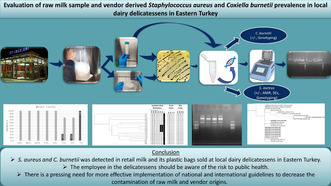

## 1 INTRODUCTION

*Staphylococcus aureus*, a widespread bacterium, establishes a commensal relationship with mammals by colonizing their cutaneous and mucosal tissues, such as skin and nasopharyngeal membranes. However, under certain conditions, this opportunistic bacterium becomes a pathogen and causes various infections in humans and animals (Martínez‐Seijas et al., 2023). *S. aureus* was recovered from various clinical specimens associated with complicated skin, endocarditis and soft tissue infections (cSSTI), pleuropulmonary infections, septic thrombophlebitis, meningitis, epidural abscesses, urinary tract infections, toxic shock syndrome (TSS), and osteoarticular (Gajdacs, 2019). In addition, *S. aureus* is the main causative agent of mastitis, a serious problem that affects the economy and public health due to the costs of veterinary care and treatment, the reduction of milk quality and quantity, the premature culling of cows, and the potential transmission to milk and dairy products (Liu et al., 2022).

*Staphylococcus aureus* can produce numerous virulence factors, including staphylococcal enterotoxins (SEs), which cause foodborne intoxication in humans and enable it to survive in the host (Gajdacs, 2019). *Staphylococcus* species secrete 24 different types of SEs, among which A, B, C, D, and E types are the most characterized (Lefebvre et al., 2022). SEs are small, soluble proteins and can withstand extreme conditions of temperature, pH, freezing, and desiccation. Due to their resistance to proteolytic degradation, SEs retain their biological activity in the gastrointestinal tract (Hennekinne et al., 2012). SEs trigger vomiting, nausea, diarrhea, and acute gastroenteritis symptoms in humans by activating emetic receptors in the intestinal mucosa (Grispoldi et al., 2019).

Multidrug‐resistant bacteria are one of the most critical challenges of the present day. The Centre for Disease Control and Prevention (USA) considers *methicillin‐resistant S. aureus* (MRSA) as a significant hazard to public health (Baran et al., 2022; Liu et al., 2022). MRSA is resistant to penicillin, cephalosporins, and carbapenems, which belong to the β‐lactam class of antibiotics. These antibiotics interfere with peptidoglycan synthesis by binding to penicillin‐binding proteins (PBPs), which are enzymes involved in cell wall formation. MRSA expresses β‐lactamases that can hydrolyze the β‐lactam ring of the antibiotics and render them inactive before they reach the PBPs. Moreover, MRSA has altered PBPs that have a low affinity for β‐lactams and can continue peptidoglycan cross‐linking. These mechanisms confer β‐lactam resistance to MRSA (Adiguzel et al., 2022; Lade & Kim, 2021). In addition, MRSA strains can be resistant to macrolides, fluoroquinolones, aminoglycosides, and clindamycin through various mechanisms (Kot et al., 2020). The development of multidrug‐resistant MRSA poses a challenge to the therapy of infections.

*Coxiella burnetii*, an obligate intracellular bacterium, is responsible for causing Q‐fever–a zoonotic disease that can contaminate a range of domestic and wild animals. Cattle, sheep, and goats are particularly susceptible to this bacterial infection. The primary means of dissemination for this disease is through inhaling aerosols that contain pathogenic bacteria, which are expelled by infected animals via their faces, urine, milk, and placental tissues (Celina & Cerný, 2022). The clinical manifestations of this zoonotic disease vary based on the duration and severity of the disease. The acute form is characterized by prolonged fever and atypical pneumonia, whereas the chronic form can cause complications including hepatitis, endocarditis, meningitis, encephalitis, and osteomyelitis (Ullah et al., 2022). *C. burnetii* may also be used as a biological weapon, which makes it a great threat to public health (Broertjes et al., 2023).

Milk is a potential vehicle for the transmission of *C. burnetii* to both humans and animals. Humans can contract the disease through multiple transmission routes, including the inhalation of contaminated dust particles and direct contact with infected animals or their products. It is imperative to exercise caution around such animals and their surroundings to prevent the spread of this disease. The bacterium has remarkable environmental resistance and can withstand pasteurization temperatures in milk. According to a recent report, there was an outbreak of Q‐fever linked to the occurrence of *C. burnetii* abortions on a dairy farm that specialized in producing artisanal cheese. The bacterium was detected in milk, cheese, and environmental samples from the farm, and serological evidence of Q‐fever was found in some farm workers and cheese consumers (Rabaza et al., 2021). Another study investigated that the bacterium was intermittently excreted in the milk serum of infected cattle and that some of the calves were infected by drinking the milk (Radinović et al., 2019). Milk contamination is often attributed to the presence of bacteria in milk‐producing animals, as well as poor hygiene practices during food production, storage, shipment, and sale.

This study aimed to investigate the contamination of retail tank milk with human‐induced *S. aureus* during the packaging stage in different enterprises and the determination and characterization of Q‐fever agent that threatens public health.

## 2 MATERIALS AND METHODS

### 2.1 Collection of samples

Two hundred samples, comprised of 100 raw milk samples and 100 bag samples, were collected from 25 local dairy delicatessens at four different time points between February and March 2022 in Erzurum, Turkey. The companies packaged the milk in transparent plastic bags and then placed it in another bag before selling it. The milk samples were transferred to the laboratory in a cold thermos, maintaining the cold chain. A plastic bag sample was collected from the surface of the transparent plastic bag using sterile swabs wetted with sterile physiological saline and placed into tubes (Becton Dickinson and Company, MD). Milk samples were transferred to sterile 50 mL falcon tubes and subjected to immediate microbiological analysis.

### 2.2 Bacteriological culture of *S. aureus*

To identify vendor‐contaminated *S. aureus*, a sterile, saline‐moistened swab was rubbed over the outer surface of a bag. For pre‐enrichment, swab and milk samples were inoculated into Nutrient Broth (Merck, Darmstadt, Germany) supplemented with horse serum and incubated under microaerophilic conditions at 37°C for 24 h. After the pre‐enrichment, a loopful of each liquid culture was streaked onto Baird Parker Medium (Lab M Limited) supplemented with egg yolk tellurite emulsion (HiMedia) and incubated at 37°C for 24 h. A pure culture was obtained from a suspicious colony that appeared black and transparent on Baird Parker Medium and then streaked on Nutrient Agar (Merck). The suspicious *S. aureus* isolates selected by Gram stain, catalase, and oxidase testing were stored at −80°C in cryogenic tubes containing Tryptone Soy Broth (Oxoid LTD) with 25% glycerol for further analysis.

The colonies, which were suspicious due to their Gram‐positive, cocci‐shaped, catalase‐positive, and oxidase‐negative characteristics, were confirmed to be *S. aureus* through the detection of the *nuc* and *femA* genes. To obtain genomic DNA, a single colony was suspended in 40 μL of single‐cell lysis buffer (SCLB, containing Tris–HCl, TE buffer, and disodium EDTA) and left in a thermocycler for 10 min at 80°C and 10 min at 55°C to lyse the bacteria. The mixture was then diluted with 80 μL of distilled water and centrifuged for 1 min at 4500 × *g* to remove cell debris (Sahin et al., 2017). For *femA* PCR (Table 1), a 25 μL PCR mix was prepared using a PCR master mix (Thermo Scientific), which contained 0.5 μL of each forward and reverse primer (10 pmol/μL). The reaction conditions were performed as previously described somewhere else (Cengiz et al., 2015). For *nuc* PCR, a 25 μL PCR mix was prepared using a PCR master mix (Thermo Scientific) containing 0.5 μL each of the *nuc*F and *nuc*R primers (10 pmol/μL) (Table 1). After heating the reaction mixture for 10 min at 94°C for pre‐denaturation, 35 cycles (60 s denaturation at 95°C, 60 s annealing at 52°C, and 60 s extension at 72°C) and 10 min post‐extension at 72°C were performed (Nagasawa et al., 2019). The products of PCR (10 μL) were run on a 1% agarose gel containing SafeViewTM Classic (Applied Biological Materials Inc.,) and 100 bp DNA ladder reference for 90 min at 75 V and 120 mA. After that, the gel was visualized under a UV transilluminator (Vilber Lourmat).

**TABLE 1:** PCR primers were used for amplification in this study.

Target genes	Primer sequence (5′‐3′)	Temperature (°C)	Size (bp)	References
*nuc*	CCTGAAGCAAGTGCATTTACGA	52	166	(Nagasawa et al., 2019)
	CTTTAGCCAAGCCTTGACGAACT			
*femA*	CTTACTTACTGCTGTACCTG	54	684	(Cengiz et al., 2015)
	ATCTCGCTTGTTATGTGC			
*mecA*	CCTAGTAAAGCTCCGGAA	54	314	
	CTAGTCCATTCGGTCCA			
*mecB*	TTAACATATACACCCGCTTG	57	279	(Becker et al., 2018)
	TAAAGTTCATTAGGCACCTCC			
*mecC*	GAAAAAAAGGCTTAGAACGCCTC	59	718	(Yilmaz et al., 2018)
	GAAGATCTTTTCCGTTTTCAGC			
*mecD*	TCCTTTAGCGATAGATGGTGAA	59	867	(Schwendener et al., 2017)
	CTCCCATCTTTTCTCCATCCT			
*sea*	GGTTATCAATGTGCGGGTGG	60	102	(Sharma et al., 2017)
	CGGCACTTTTTTCTCTTCGG			
*seb*	GTATGGTGGTGTAACTGAGC	60	164	
	CCAAATAGTGACGAGTTAGG			
*sec*	AGATGAAGTAGTTGATGTGTATGG	60	451	
	CACACTTTTAGAATCAACCG			
*sed*	CCAATAATAGGAGAAAATAAAAG	60	278	
	ATTGGTATTTTTTTTCGTTC			
*Trans*1	TATGTATCCACCGTAGCCAGTC	60	687	(Youssef et al., 2021)
*Trans*2	CCCAACAACACCTCCTTATTC			

### 2.3 Enterotoxin gene detection

SEA, SEB, SEC, and SED loci from the extracted DNA of *S. aureus* isolates were determined by multiplex PCR using specific primers (Table 1). A 25 μL PCR mix was prepared using a PCR master mix (Thermo Scientific) containing 0.5 μL of each primer (10 pmol/μL). After heating the reaction mixture for 15 min at 95°C for pre‐denaturation, 35 cycles (60 s denaturation at 91°C, 60 s annealing at 60°C, and 60 s extension at 72°C) and 10 min post‐extension at 72°C were performed (Sharma et al., 2017). PCR products (10 μL/sample) were run on a 1% agarose gel containing SafeViewTM Classic (Applied Biological Materials Inc.) and 100 bp DNA ladder reference for 90 min at 75 V and 120 mA. After gel electrophoresis, the gel was visualized under a UV transilluminator (Vilber Lourmat).

### 2.4 Antimicrobial susceptibility test

The antimicrobial resistance of the confirmed *S. aureus* was determined using the disc diffusion method according to the EUCAST (European Committee on Antimicrobial Susceptibility Testing) standards (EUCAST, 2023). Antibiotics (Oxoid) were selected from three classes of antibiotics, including oxacillin (1 μg), penicillin (10 μg), cefoxitin (30 μg), ampicillin/sulbactam (20 μg), amoxicillin/clavulanic acid (30 μg), ceftiofur (30 μg), tetracycline (30 μg), gentamicin (10 μg), and oxytetracycline (30 μg).

### 2.5 Detection of methicillin‐resistant *S. aureus*

The phenotypic resistance of *S. aureus* isolates was investigated by the disk diffusion method using EUCAST criteria (EUCAST, 2023). Isolates that were resistant to cefoxitin (30 μg) in the disk diffusion method were considered phenotypically resistant to methicillin. The *S. aureus* ATCC 29213 was used as a reference strain in the experiments.

### 2.6 Repetitive sequence‐based PCR (rep‐PCR) fingerprinting

The genotypic methicillin resistance of the isolates was investigated by the detection of *mec* genes (*mec*A, *mec*B, *mec*C, and *mec*D) through PCR (Horie et al., 2009). The primer sequences and annealing temperature for amplification of the *mec* genes of the isolates are shown in Table 1. PCR mix and electrophoresis were performed as previously described above in this study. The genetic diversity between *S. aureus* isolates was detected by rep‐PCR. The rep‐PCR was performed with primer (GTG)_5_ on the isolates extracted as previously described (Baran et al., 2022; Cengiz et al., 2023). A 25 μL PCR mix was prepared using a PCR master mix (Thermo Scientific) containing 1 μL of (GTG)_5_ primer. After heating the reaction mixture for 7 min at 94°C for pre‐denaturation, 30 cycles (60 s denaturation at 94°C, 60 s annealing at 40°C, and 8 min extension at 65°C) and 16 min post‐extension at 65°C were performed (Švec et al., 2010). Electrophoresis and imaging were performed on a 1% agarose gel containing SafeViewTM Classic (Applied Biological Materials Inc.). The obtained rep‐PCR fingerprints were analyzed according to the presence or absence of bands. The bands' data were exported to Microsoft Excel to create a data matrix (Albufera et al., 2009). The matrix data were analyzed using the unweighted pair group method with the arithmetic mean (UPGMA) algorithm. Relationships between various band patterns are visualized by ITOL online (Baran et al., 2022).

### 2.7 *Coxiella burnetii* identification and genotyping

Genomic DNA was recovered from raw milk samples using a commercial kit (GeneJET Genomic DNA Purification Kit, Thermo Scientific), and the presence of *C. burnetii* was confirmed by PCR with specific primers. For DNA extraction, 50 mL of milk samples were centrifuged at 2200 × *g* for 10 min. At the end of centrifugation, the upper‐fat layer and liquid were discarded. To dissolve the milk casein, the pellet was suspended by adding 200 μL of TE (10 mM Tris–HCl, 1 mM EDTA, pH 7.6) and 300 μL of 0.5 M EDTA (pH 8.0), then transferred into a 1.5 mL sterile centrifuge tube at 3000 × *g* for 10 min to form a pellet. After the pellet was diluted with 200 μL of PBS, DNA isolation was performed using the DNA purification kit (Thermo Fisher Scientific). Trans‐1 and Trans‐2 primers specific to the IS1111 transposase gene region (Table 1) targeting the repetitive transposon‐like region of *C. burnetii* were used for identification. For DNA amplification, pre‐denaturation at 95°C for 5 min, 35 cycles (30 s denaturation at 95°C, 30 s annealing at 60°C, and 60 s extension at 72°C), and a 10 min post‐extension at 72°C were performed (Günaydin et al., 2015). 1% agarose gel containing SafeViewTM Classic (Applied Biological Materials Inc.) for 90 min at 75 V and 120 mA was used to visualize PCR products. The gel was visualized under a UV transilluminator (Vilber Lourmat). The 687‐bp‐long band was evaluated as positive for *C. burnetii*. Sanger sequencing was performed by randomly selecting seven samples with positive PCR results. Samples having sequences between 570 and 663 bp were sent for Sanger sequencing. Gene alignment of these sequences was performed with CHROMAS programs. A phylogenetic dendrogram was created for twenty different sequences from different countries, including other Turkey strains, using the MEGA (version 10.2.6) program. The results were analyzed using the maximum likelihood method, phylogenetic tree (Kimura 2‐parameter method), and bootstrapping analysis (1,000 replicates). The results of the partial sequence of the transposase gene were deposited in NCBI GenBank.

### 2.8 Statistical analysis

Results were calculated using descriptive statistics, including the mean and standard deviation. The distribution of the values for each variable (positivity of *C. burnetii*) and in each group (sample origin) was tested using the D'Agostino‐Pearson normality test; depending on the result, the significance of differences between parameters was determined via an unpaired *t* test. *p* < .05 was taken as statistical significance. Statistical analyses were performed using SPSS (SPSS Inc).

## 3 RESULTS

### 3.1 Isolation, identification, enterotoxin genes, antimicrobial resistance, and dendrogram analysis of *S. aureus*

In total, 100 retail milk and 100 plastic bag swab samples in eastern (Erzurum province) Turkey between February and March 2022 were used in this study. The suspicious *S. aureus* strains in terms of microscopic morphology (Gram staining), catalase, and oxidase tests were isolated from 32 of 100 milk samples and 28 of 100 plastic bags with human contact. The *nuc* and *femA* genes were detected in 8 of 100 (8%) retail milk samples and in 14 of 100 (14%) isolates from plastic bag samples. Out of 200 retail milk and plastic bag samples, 22 (11.0%) samples were isolated and confirmed as *S. aureus* (*p* > .05).

The *S. aureus* strains isolated in the current study were screened to detect enterotoxin genes by PCR. The enterotoxin gene results showed that none of the isolated *S. aureus* strains from milk samples or plastic bags contained SEA, SEB, SEC, and SED genes.

**FIGURE 1:**
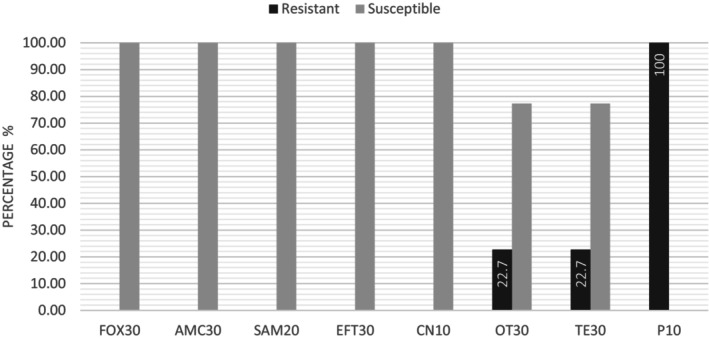
Antimicrobial susceptibility profiles of 22 *S. aureus* strains isolated from retail milk and plastic bag samples. AMC, Amoxicillin Clavulanic Acid; CN, Gentamycin; EFT, Ceftiofur; FOX, Cefoxitin; OT, Oxytetracycline; P, Penicillin G; SAM, Sulbactam Ampicillin; TE, Tetracycline.

The antimicrobial susceptibility test using the disk diffusion method following the EUCAST instruction was performed for a total of 22 (11.0%) *S. aureus* strains isolated from retail milk and plastic bag samples. Twenty‐two *S. aureus* strains were found to be sensitive to ceftiofur, cefoxitin, ampicillin sulbactam, amoxicillin‐clavulanic acid, and gentamicin. However, 22.7% (5/22) of *S. aureus* strains were resistant to tetracycline and oxytetracycline, and all isolated *S. aureus* strains (100.0%) were resistant to penicillin G (Figure 1). The strains under observation do not meet the criteria for multi‐drug resistance as they display susceptibility to no more than three classes of antibiotics.

To reveal the phenotypic methicillin resistance in *S. aureus* strains, cefoxitin disk diffusion (30 μg) was performed, whereas, for the genotypic identification of methicillin resistance, PCR was used for the detection of *mec* genes. Of the 22 strains identified and confirmed as *S. aureus*, none of the strains were both phenotypically and genotypically identified as MRSA.

**FIGURE 2:**
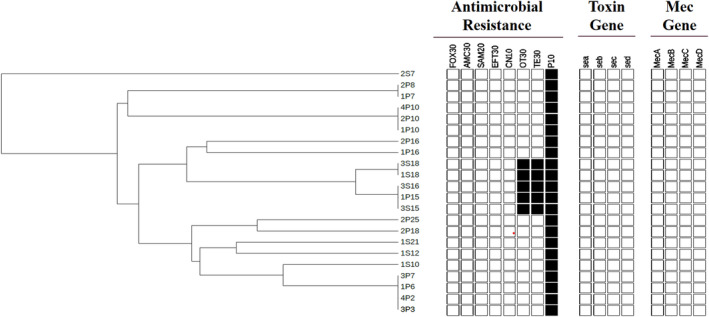
The band patterns based on rep‐PCR dendrogram of *S. aureus* isolates from retail milk and plastic bag samples. The generation of a data matrix depicting antimicrobial susceptibility patterns, toxin genes, and *mec* genes, was performed through the use of Microsoft Excel. The matrix clearly indicates the presence (black box) or absence (empty box) of the aforementioned. AMC, Amoxicillin Clavulanic Acid; CN, Gentamycin; EFT, Ceftiofur; FOX, Cefoxitin; OT, Oxytetracycline; P, Penicillin G; SAM, Sulbactam Ampicillin; TE, Tetracycline.

In the (GTG)_5_ fingerprint (rep‐PCR), isolates exhibited 7–11 bands ranging from 700 bp to 3600 bp. Two main clusters were formed in the dendrogram analysis. One of them is divided into three separate clusters, including most of the strains (Figure 2). These clusters consisted of five groups with 100% similarity to each other and 8 independent isolate groups.

### 3.2 Detection and Sanger sequencing of *C. burnetii*

**FIGURE 3:**
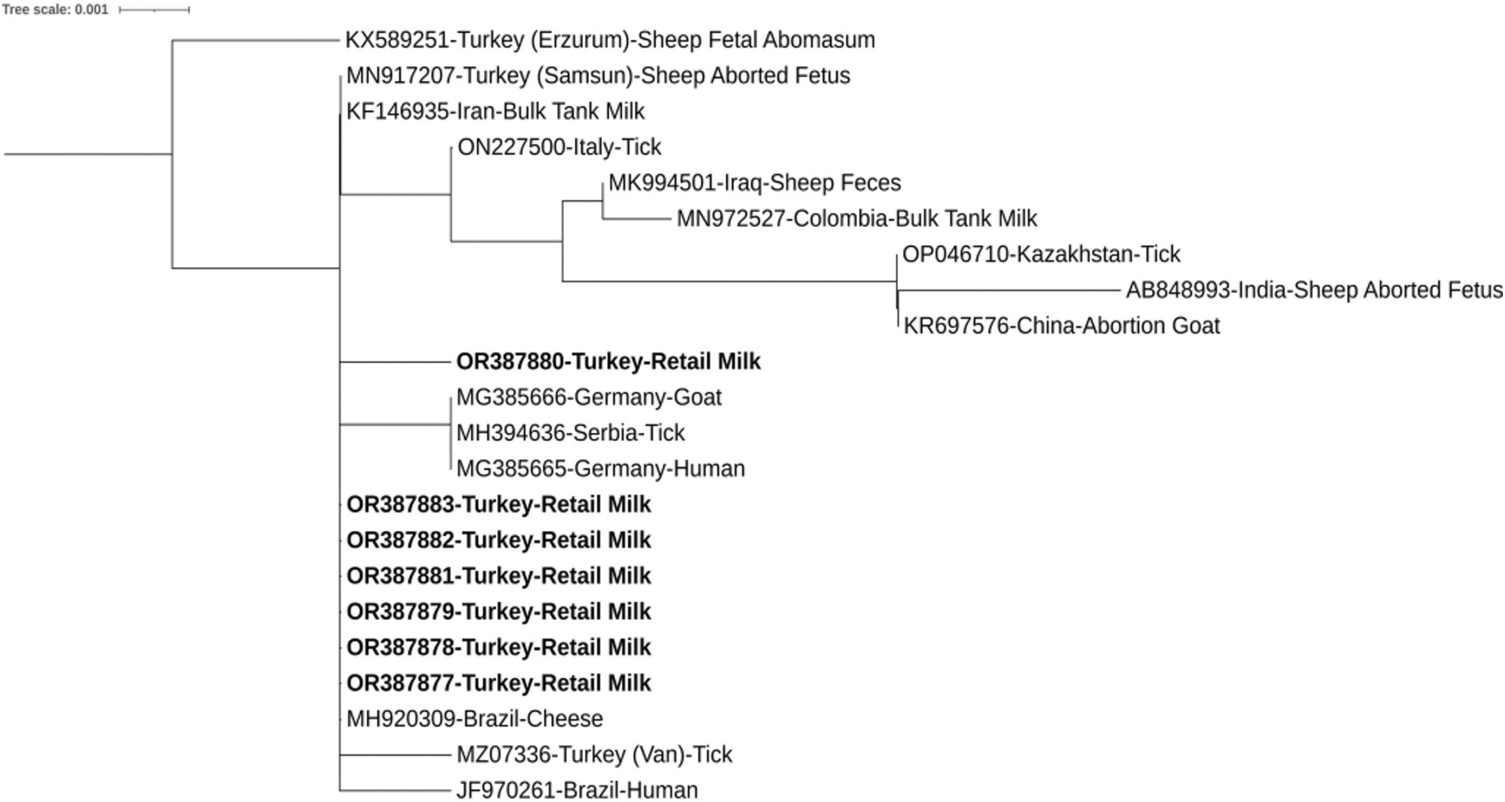
The Maximum Likelihood method and the Tamura‐Nei model were utilized to create the phylogenetic tree of *C. burnetii*. A consensus tree was formed from 1000 replicates to represent the analyzed taxa. Branches that were reproduced in less than 50% of bootstrap replicates were removed. The phylogenetic tree involved 22 sequences, with a total of 626 positions in the final dataset. MEGA X was used to conduct the phylogenetic analyses. The nucleotide sequences used in this study are indicated in bold.

The presence of *C. burnetii* in retail milk samples was screened by PCR using primers specific to the IS1111 transposase gene region. The *C. burnetii* DNA was determined in 14.0% (*n* = 100) of retail milk samples (*p* > .05). The six retail milk samples were positive in the first sampling, three of them were second and third, and two of them were positive in the last sampling. Sanger sequencing was performed on seven randomly selected *C. burnetii*‐positive PCR products. The strains matched 100% with the *Coxiella brunetii* RSA 493 reference strains in the NCBI GenBank database. We deposited it in GenBank with accession numbers from OR387877 to OR387883. For phylogenetic comparison, the nucleotide sequences of 22 *C. burnetii* were selected from the NCBI GenBank database according to data size, countries, isolation source, and host. The phylogenetic tree displayed two main clades. The phylogenetic tree indicated that six of the *C. burnetii* sequences from the current study were genetically distinct from the other *C. burnetii* sequences previously reported from Turkey (MN868465, MZ073364, and KX589251), whereas they were in the same cluster as a sequence from Brazil (MH920309). Interestingly, one of the sequences (OR387880‐Turkey‐Retail Milk) in this study was placed far from the sequences reported from Turkey (KX589251 and MN917207), indicating less similarity (Figure 3).

## 4 DISCUSSION

*Staphylococcus aureus* and *C. burnetii* can be transmitted from animals to humans and have a significant impact on both veterinary and public health worldwide (Youssef et al., 2021). These bacteria can adversely affect the livestock industry. *S. aureus* frequently causes foodborne illness in humans, whereas *C. burnetii* is responsible for Q‐fever (Mangombi‐Pambou et al., 2023; Savini et al., 2023). By scrutinizing the epidemiology and genomics of these pathogens from diverse sources, we can obtain a more in‐depth understanding of their potential, transmission patterns, and evolutionary connections.

In this study, we investigated the occurrence of *S. aureus* in retail milk and plastic bags containing retail milk, as well as *C. burnetii* in retail milk samples collected from February to March 2022 in eastern Turkey. All 22 (11.0%) *S. aureus* strains, 8 from retail milk and 14 from plastic bag samples with human contact, were confirmed through biochemical tests and PCR. The similar prevalence of *S. aureus* in retail milk samples is supported by previous studies conducted in Turkey and other countries (Güneş et al., 2022; Keyvan, 2019; Kou et al., 2021; Lemma et al., 2021). In a meta‐analysis and systematic review, the prevalence of *S. aureus* in raw retail milk was reported as 33.36% (95% CI: 27.18%–39.84%), further emphasizing a significant level of contamination of milk with *S. aureus* and the threat of public health (Zhang et al., 2022). The antimicrobial resistance of *S. aureus* strains in the present study revealed that all strains were resistant to penicillin G, even though a low level of resistance against tetracycline and oxytetracycline (22.7%) was detected. Similar to our study, the highest level of resistance has been reported for penicillin and tetracycline antibiotics in previous studies by Günaydin et al. (2015) and Chenouf et al. (2021). The β‐lactam class of antibiotics is often used in Turkey to treat staphylococcal mastitis in dairy cattle (Yilmaz et al., 2018). Tetracycline is frequently used for treating genitourinary infections, septicemia, digestive infections, respiratory infections, and interdigital infections in cattle (Filazi & Yurdakok, 2010; Yilmaz et al., 2018). Considering that these antibiotics belong to old families of drugs prescribed for treatment or prophylaxis in dairy cattle, their continued use and selective pressure may largely explain the high rates of resistance. Molecular analysis of the 22 isolated *S. aureus* strains indicated that none of the strains harbored either *mec* genes (*mecA, mecB, mecC*, and *mecD*) or enterotoxin genes (SEA, SEB, SEC, and SED). Similar results to our research were found in a study conducted by Crago et al. (2012). In contrast to our findings, the *mecA* gene harboring *S. aureus* isolated from raw milk samples was reported in Ethiopia (5.0%), China (21.0%), Algeria (3.6%), and Turkey (2.79% and 5.0%) (Baran et al., 2022; Chenouf et al., 2021; Günaydin et al., 2015; Kou et al., 2021; Siriken et al., 2018). The absence of *mec* genes in the *S. aureus* strains observed in this study may be due to antibiotic‐related factors, differences in study design, limited sample size, or specimen types tested. Interestingly, rep‐PCR analysis revealed that retail milk and plastic bag surface *S. aureus* strains from the same company shared the same genotypic characteristics, indicating that human hand contact plays a role in the transmission of bacteria. This finding further emphasizes that hand hygiene should be adopted by people working in retail shops.

In our study, 14 (14%) retail milk samples were positive for *C. burnetti*, and 100% matched the *Coxiella brunetii* RSA 493 reference strains in the GenBank database. The phylogenetic tree based on the transposase gene of *C. burnetti* showed less genetic diversity among strains from different countries, as shown in Figure 3. The overall low positivity for *C. burnetii* observed in the current study is comparable to previously reported studies, with a positivity of 6% in bovine milk samples in Turkey (Can et al., 2015), and the lower PCR positivity (1.42% and 0.4%) has also been reported in cattle and sheep milk samples in Turkey, respectively (Saglam & Sahin, 2016). Furthermore, 39 caprine, 81 ovine, and 359 bovine bulk milk samples were analyzed in Switzerland, and 4.7% of *C. burnetii* positivity was detected only in bovine bulk milk samples (Fretz et al., 2007). Another study in dairy cattle from the Setif province of Algeria tested raw milk samples, and the results highlighted that 9% of the samples contained *C. burnetii* (Menadi et al., 2022). In addition, raw milk samples from sheep and goats in West Azerbaijan province, Iran, were analyzed using nested PCR, and the results showed that 51 (12.1%) samples from sheep and goats were positive for *C. burnetii* (Khademi et al., 2020). A study conducted on dairy cows in France revealed 24.4% of milk samples carried *C. burnetii* (Guatteo et al., 2006). In contrast to our findings, 94.3% *C. burnetii* positivity was detected from bulk tank milk samples in the USA (Kim et al., 2005). A recent study in Italy recommended that microbiological criteria for raw milk should be revised to include the detection of *C. burnetii*, particularly when the milk in question is intended for human consumption (Petruzzelli et al., 2013). These findings may be relevant to individuals or organizations involved in the production and distribution of raw milk products. These findings further indicate that the quality of raw milk should be assessed and the effectiveness of milk pasteurization methods should be measured in milk processing corporations worldwide to ensure consumer health protection.

## 5 CONCLUSION

In this study, *S. aureus* was detected in retail milk and its plastic bags sold at local dairy delicatessens in Eastern Turkey. The *C. burnetii* was only revealed from retail milk samples. The findings from the current study indicate that raw milk may trigger very severe illnesses in humans. Employees in the delicatessens should be aware of the risk to public health. As such, closer scrutiny of foodborne bacteria contamination at dairy sources and vendors is necessary, and a comprehensive understanding of the transmission routes and potential infection sources is critical. Furthermore, there is a pressing need for more effective implementation of national and international guidelines to decrease the contamination of raw milk and vendor origins.

## AUTHOR CONTRIBUTIONS

**Muhammed Furkan Kaplan:** Methodology (equal); software (equal); visualization (equal). **Ece Kaplan:** Methodology (equal); software (equal); visualization (equal). **Ali Raza:** Data curation (equal); investigation (equal). **Mehtap Demirler:** Methodology (equal); software (equal); visualization (equal). **Alper Baran:** Data curation (equal); formal analysis (equal); investigation (equal); validation (equal); writing – original draft (equal). **Seyda Cengiz:** Methodology (equal); software (equal); visualization (equal). **Mehmet Cemal Adiguzel:** Conceptualization (equal); data curation (equal); methodology (equal); project administration (equal); resources (equal); supervision (equal); writing – original draft (equal); writing – review and editing (equal).

## CONFLICT OF INTEREST STATEMENT

The authors declare that they have no conflicts of interest in this article.

## Data Availability

The data that support the findings of this study are available from the corresponding author upon reasonable request.
